# Update on Resection Strategies for Hepatocellular Carcinoma: A Narrative Review

**DOI:** 10.3390/cancers16234093

**Published:** 2024-12-06

**Authors:** Jun Suh Lee, Hyeong Woo Choi, Ji Su Kim, Tae Yoon Lee, Young Chul Yoon

**Affiliations:** Department of Surgery, Incheon St. Mary’s Hospital, College of Medicine, The Catholic University of Korea, Seoul 06591, Republic of Korea; rudestock@gmail.com (J.S.L.);

**Keywords:** HCC, hepatectomy, surgical outcomes

## Abstract

Hepatocellular carcinoma (HCC) is the most common primary liver cancer, the incidence of which is rising globally. Despite recent advancements in immunotherapeutic and surgical treatment modalities, the prognosis for HCC remains poor. The surgical treatment strategy for HCC comprises a multimodal effort that ranges from ablative therapy and surgical resection to liver transplantation. Thanks to collective efforts from the surgical society, there have been rapid advances in resection strategies, such as 3D printing for surgical planning and minimally invasive techniques to minimize surgical trauma. This review examines recent advancements in surgical techniques, patient selection criteria, and perioperative management for HCC resection. The purpose of this review was to provide clinicians and researchers with an up-to-date perspective on the evolving role of surgical resection in HCC treatment, and to identify key areas for future investigation to improve patient outcomes.

## Simple Summary

This review focuses on recent advancements in the surgical treatment of hepatocellular carcinoma (HCC), the most common type of primary liver cancer. Despite improvements in immunotherapy and surgery, HCC still has a poor outlook. This review discusses various surgical approaches and highlights new developments like 3D printing for surgical planning and minimally invasive techniques that reduce surgical trauma. The review covers recent progress in surgical methods, how patients are selected for surgery, and how they are cared for before and after the operation. Its goal is to update doctors and researchers on the changing role of surgery in treating HCC and to point out areas where more research could lead to better results for patients.

## 1. Introduction

### 1.1. Brief Overview of Hepatocellular Carcinoma

Hepatocellular carcinoma (HCC) ranks as the third deadliest form of cancer worldwide, and only about 18% of patients survive five years after diagnosis [1]. Major risk factors are hepatitis B and C, alcohol abuse, nonalcoholic fatty liver disease (NAFLD), and exposure to toxins like aflatoxins [2,3]. Cirrhosis remains the most significant risk factor, regardless of etiology [4]. Since 1990, there has been a notable shift from virus-caused liver disease to non-viral causes. While hepatitis B virus (HBV) was responsible for over 50% of cases in 1990, this dropped to 42% by 2019. Meanwhile, alcohol-related liver disease increased from 13% to 18%, and NAFLD rose from 5% to 6% during this same period [5,6].

The incidence of HCC has been rising globally, particularly in Western countries, largely due to the increasing prevalence of obesity and metabolic syndrome. Early detection through surveillance can improve outcomes, but it is underutilized, even in developed countries [2]. HCC presents unique challenges in diagnosis and management due to the underlying liver dysfunction often present in affected patients. The disease is characterized by its heterogeneity in presentation, molecular profiles, and treatment responses, necessitating a multidisciplinary approach to patient care.

Treatment options range from curative approaches such as liver transplantation (LT) and surgical resection to palliative measures such as chemoembolization and targeted therapies, depending on disease stage and liver function [2,4].

### 1.2. Importance of Surgical Resection in HCC Treatment

Surgical resection remains a crucial treatment option for HCC, offering the potential for cure in selected patients [7,8]. It is typically considered the first-line treatment for patients with early-stage HCC and preserved liver function. Advances in surgical techniques, perioperative care, and patient selection have improved survival rates, with 5-year survival ranging from 40 to 70% [9,10].

The importance of surgical resection in HCC treatment is underscored by several factors. Firstly, it offers curative intent, aiming to completely remove the tumor and providing the best chance for long-term survival. Modern surgical techniques allow for the removal of tumor tissue while maximizing the preservation of functional liver parenchyma, which is crucial for patients with underlying liver disease. Anatomic resection is generally preferred over non-anatomic resection for better oncologic outcomes [11]. However, the extent of resection must balance tumor removal with the preservation of functional liver tissue [7].

Resection also provides complete pathological specimens, allowing for detailed histological and molecular analysis, which can guide further treatment decisions and prognostication. In some cases, resection can serve as a bridge to LT, buying time for patients awaiting donor organs. Even in cases of tumor recurrence, repeat resection may be possible, offering continued disease control.

Despite these advantages, challenges remain in the surgical management of HCC. High recurrence rates continue to be a significant issue [9]. While current guidelines limit resection to early-stage HCC, some argue for expanding indications to include more advanced cases [12]. Multimodal approaches combining resection with other therapies may improve prognosis [8]. For patients ineligible for resection, LT or ablation may be considered [10].

The role of surgical resection in HCC management continues to evolve. Ongoing research focuses on refining patient selection criteria, improving surgical techniques, and integrating resection with other treatment modalities to optimize outcomes for patients with HCC.

### 1.3. Aim and Scope of This Review

This narrative review aims to provide a comprehensive update on current resection strategies for HCC. This review will examine recent advancements in surgical techniques and technologies for HCC resection, discussing evolving patient selection criteria and preoperative evaluation methods. It will explore the integration of resection with other treatment modalities in multimodal approaches and analyze outcomes and prognostic factors associated with different resection strategies.

Special clinical scenarios and their impact on resection decisions will be considered, along with emerging trends and future directions in the surgical management of HCC. By addressing these aspects, this review seeks to offer clinicians and researchers an up-to-date perspective on the role of resection in HCC treatment, aiding in clinical decision making and identifying areas for future research and improvements in patient care.

## 2. Current Guidelines for HCC Resection

HCC management, particularly the role of surgical resection, has been the subject of extensive research and debate in recent years. To standardize care and improve outcomes, several international organizations have developed comprehensive guidelines for HCC treatment. These guidelines, while sharing many commonalities, also reflect regional differences in disease prevalence, available resources, and clinical experience. This section will explore the major international guidelines for HCC resection and discuss the key criteria used for patient selection.

### 2.1. Major International Guidelines

Several international organizations have developed guidelines for the management of HCC, including recommendations for surgical resection. While these guidelines share many commonalities, they also have distinct nuances, reflecting regional differences in HCC epidemiology, available resources, and clinical experience. The most prominent and widely referenced guidelines are as follows.

A. European guidelines

The European Association for the Study of the Liver (EASL) guidelines, updated in 2018, provide comprehensive recommendations for HCC management in Europe [13]. These guidelines emphasize the importance of multidisciplinary team decisions and provide specific criteria for resection candidacy. EASL guidelines are generally more conservative in their resection criteria compared to Asian guidelines. They recommend resection for single tumors without macrovascular invasion in patients with preserved liver function and without clinically significant portal hypertension. However, the EASL guidelines mean that experienced centers have reported acceptable outcomes for patients, exceeding conventional criteria. Assessment tools such as The Model for End-Stage Liver Disease (MELD) score and its sodium-adjusted version (MELD-Na), along with indocyanine green retention testing, liver stiffness measurements (LSMs), and the cholinesterase to bilirubin ratio, have proved valuable in expanding selection criteria for patients with borderline liver function [14,15,16].

The Barcelona Clinic Liver Cancer (BCLC) staging system and treatment strategy, while not a guideline per se, is widely used and referenced in many guidelines, particularly in Europe and North America [14]. It provides a framework for linking staging with treatment recommendations, including resection. The BCLC system recommends resection only for very early (0) and early (A) stage HCC in patients with preserved liver function and without portal hypertension. This conservative approach has been challenged by some experts, particularly in Asia, who argue for expanded resection criteria.

B. American Guidelines

The American Association for the Study of Liver Diseases (AASLD) guidelines, last updated in 2018, offer recommendations tailored to the North American context [15]. These guidelines provide a framework for diagnosis, staging, and treatment of HCC, including specific indications for resection. AASLD guidelines are similar to EASL in their conservative approach to resection. They recommend resection for single tumors in patients with Child-Pugh A cirrhosis and without portal hypertension. However, AASLD places greater emphasis on the use of LT when possible, positing that LT is the treatment of choice for patients with portal hypertension and hepatic decompensation with early-stage HCC within Milan criteria (1 tumor up to 5 cm, or two to three tumors with the largest being <3 cm) [16]. This reflects the greater availability of donor organs in the United States.

The National Comprehensive Cancer Network (NCCN) guidelines, updated annually, provide a consensus view from major cancer centers in the United States [17]. These guidelines offer a detailed algorithm for HCC management, including specific recommendations for when to consider resection. NCCN guidelines are generally aligned with AASLD but provide more detailed recommendations for various clinical scenarios. They emphasize the role of multidisciplinary tumor boards in decision making and are more open to considering resection in patients with minimal comorbidities and limited portal hypertension.

C. Asian Guidelines

The Asian Pacific Association for the Study of the Liver (APASL) guidelines, updated in 2017, are particularly relevant for Asian countries, where HCC incidence is high [18]. Liver resection is indicated for more progressed HCC in terms of tumor burden in the treatment algorithms of Asian countries, reflecting the greater experience with this procedure in Asia [19]. APASL guidelines allow for resection in select patients with multiple tumors and mild portal hypertension, provided there is sufficient future liver remnant. They also place greater emphasis on the use of the indocyanine green retention rate at 15 min (ICG-15) for assessing liver function, a test not commonly used in Western countries.

The Japan Society of Hepatology (JSH) guidelines, while less widely used internationally, offer valuable insights given Japan’s extensive experience with HCC management [20]. These guidelines are more aligned with APASL in their approach, allowing for more aggressive resection strategies. For example, hepatectomy is offered as a viable option in selected patients with portal hypertension [21]. They use a unique scoring system that incorporates liver function measures (including ICG-15), tumor characteristics, and portal hypertension to guide treatment decisions.

In summary, while all guidelines agree on the importance of preserved liver function and limited tumor burden for resection candidacy, the Asian guidelines (APASL and JSH) tend to be more permissive in their resection criteria compared to Western guidelines (EASL, AASLD, and NCCN). This reflects a variety of factors, such as differences in HCC epidemiology, surgical expertise, and transplant donor availability. Understanding these nuances is crucial for clinicians in interpreting and applying these guidelines in their practice. The key differences of each guideline are outlined in Table 1.

### 2.2. Patient Selection Criteria

While there are some variations among guidelines, the general criteria for selecting patients for HCC resection can be summarized as follows:

1. Tumor characteristics: Most guidelines recommend resection for single tumors without macrovascular invasion. The EASL and AASLD guidelines generally limit resection to single tumors of any size, while the APASL guidelines are more permissive, considering resection for multiple tumors if technically feasible.
2. Liver function: All guidelines emphasize the importance of preserved liver function. This is typically assessed using the Child-Pugh score, with Child-Pugh A patients being the best candidates. Some guidelines, particularly APASL, also recommend the use of the indocyanine green retention rate at 15 min (ICG-15) to assess functional liver reserve.
3. Portal hypertension: The EASL and AASLD guidelines generally recommend against resection in patients with clinically significant portal hypertension, defined as a hepatic venous pressure gradient ≥ 10 mmHg or the presence of varices. However, the APASL guidelines are less strict on this criterion.
4. Performance status: Good performance status is required across all guidelines, typically defined as Eastern Cooperative Oncology Group (ECOG) performance status 0–1.
5. Remnant liver volume: All guidelines stress the importance of ensuring adequate remnant liver volume after resection, although specific volumetric criteria vary.

It is important to note that these criteria are general guidelines, and individual patient factors, as well as center expertise, play crucial roles in the final decision for resection. Many guidelines emphasize the importance of multidisciplinary team discussions in making these complex decisions.

Recent evidence suggests that liver resection may be safely performed in selected HCC patients who fall outside conventional guidelines. In a single-center study of 150 patients by Barros et al., those with one BCLC contraindication factor achieved median overall and disease-free survival of 43.3 and 15.1 months, respectively [22]. Had the BCLC 2010 and 2018 guidelines been strictly followed, 46.7% and 26.7% of patients, respectively, would have been denied potentially curative treatment. Similarly, Lu et al. demonstrated, in a larger cohort of 556 surgical patients, that hepatectomy was feasible in those with clinically significant portal hypertension (CSPH) beyond the Milan criteria [23]. While these patients had inferior survival compared to those within Milan criteria, their overall survival was significantly better than the 172 patients who received Transarterial Chemoembolization (TACE). Notably, there was no significant difference in 90-day mortality and complications between minor and major hepatectomy in patients beyond Milan criteria. These findings suggest that carefully selected patients with traditionally considered contraindications may still benefit from surgical resection, though those with multiple adverse factors should be evaluated more cautiously.

### 2.3. Conclusions

In summary, the current guidelines for HCC resection emphasize the importance of careful patient selection based on tumor characteristics, liver function, and overall health status. While there are some variations among guidelines, particularly between Western and Asian approaches, they generally agree on the following key points:

1. Resection is primarily recommended for single tumors without macrovascular invasion.
2. Preserved liver function, typically assessed by Child-Pugh score, is crucial for resection candidacy.
3. The presence of significant portal hypertension is generally considered a contraindication to resection in Western guidelines, though Asian guidelines are more permissive.
4. Good performance status is required across all guidelines.
5. Adequate future liver remnant must be ensured to prevent post-hepatectomy liver failure.

Despite these guidelines, the decision to proceed with resection often requires individualized assessment and multidisciplinary discussion, taking into account center expertise and patient preferences. As our understanding of HCC biology and surgical techniques continues to evolve, it is likely that these guidelines will be further refined to optimize patient outcomes.

## 3. Pre-Operative Evaluation

Pre-operative evaluation is a critical step in the management of patients with hepatocellular carcinoma (HCC) who are being considered for surgical resection. This evaluation encompasses liver function assessment and tumor staging. Both of these components play a crucial role in determining the feasibility and safety of resection, as well as in planning the surgical approach.

### 3.1. Liver Function Assessment

Liver function assessment is crucial in determining whether a patient can tolerate resection and the extent of resection that can be safely performed. The Child-Pugh score remains the most widely used tool for assessing liver function in HCC patients [24]. It incorporates five parameters: serum bilirubin, serum albumin, prothrombin time, ascites, and hepatic encephalopathy. Generally, only patients with Child-Pugh A cirrhosis are considered for major resections. However, the Child-Pugh score has limitations, particularly in differentiating among patients within the Child-Pugh A category. Therefore, additional tools are often employed. The MELD score, originally developed to predict survival in patients undergoing transjugular intrahepatic portosystemic shunt procedures, is now widely used in LT and has also shown value in predicting post-hepatectomy liver failure [25]. In Asian countries, particularly Japan, the ICG-R15 is commonly used to assess liver function and determine the safe extent of liver resection [26]. Portal hypertension, typically defined as a hepatic venous pressure gradient ≥ 10 mmHg, is an important consideration, as it is associated with an increased risk of postoperative liver decompensation. While direct measurement via hepatic vein catheterization is the gold standard, non-invasive indicators, such as the presence of esophageal varices or splenomegaly with thrombocytopenia, are often used as surrogate markers [27].

Furthermore, advanced imaging modalities have significantly enhanced the assessment arsenal, including Tc-99m-labeled galactosyl serum albumin scintigraphy and gadolinium-enhanced magnetic resonance imaging, which provide detailed functional liver volume analysis [28]. Non-invasive techniques for evaluating liver fibrosis and portal hypertension have also emerged, utilizing liver stiffness measurements through ultrasonography or magnetic resonance elastography, complemented by non-invasive fibrosis markers.

While it is determined as the single best method for evaluating liver function ahead of liver resection, this comprehensive evaluation strategy has become essential for optimizing surgical outcomes and minimizing PHLF risk in patients undergoing liver resection.

### 3.2. Tumor Staging

Tumor staging is essential not only for prognostication but also for treatment planning. While several staging systems exist for HCC, the BCLC staging system is the most widely used and endorsed by major Western guidelines [14]. It integrates tumor burden, liver function (Child-Pugh score), and performance status to guide treatment decisions. According to the BCLC system, resection is typically recommended for patients with single tumors, preserved liver function, and absence of clinically significant portal hypertension. Another widely used staging system is the modified Union for International Cancer Control (UICC) system. In 1996, Izumi et al. proposed a modification to the UICC staging system, concluding that the original UICC system did not have any prognostic significance [29]. The superiority of the modified UICC system was validated in an Italian cohort [30]. Currently, the modified UICC staging system is being widely used as one of the main staging systems for HCC. In clinical practice, staging for potential resection candidates focuses on assessing tumor number, size, location, vascular invasion, and extrahepatic spread. Positron emission tomography (PET) with fluorodeoxyglucose (FDG) can be useful in detecting extrahepatic metastases, although its sensitivity for intrahepatic HCC is limited due to the variable FDG avidity of these tumors [31].

In conclusion, comprehensive pre-operative evaluation integrating liver function assessment and appropriate tumor staging is crucial for selecting suitable candidates for HCC resection and for optimizing surgical outcomes. This multifaceted approach allows for personalized treatment planning, balancing the potential benefits of resection against the risks of postoperative complications and liver failure.

## 4. Resection Strategies

Surgical resection remains a cornerstone in the treatment of HCC, offering the potential for a cure in selected patients. The choice of resection strategy is crucial and depends on various factors, including tumor characteristics, liver function, and patient status. This section explores five key aspects of HCC resection strategies: anatomical versus non-anatomical resection, parenchyma-sparing techniques, extended hepatectomy considerations, laparoscopic versus open approaches, and robotic-assisted surgery. As our understanding of HCC biology and surgical techniques continues to evolve, so too do our approaches to surgical resection of this challenging disease.

### 4.1. Anatomical vs. Non-Anatomical Resection

**Figure 1:**
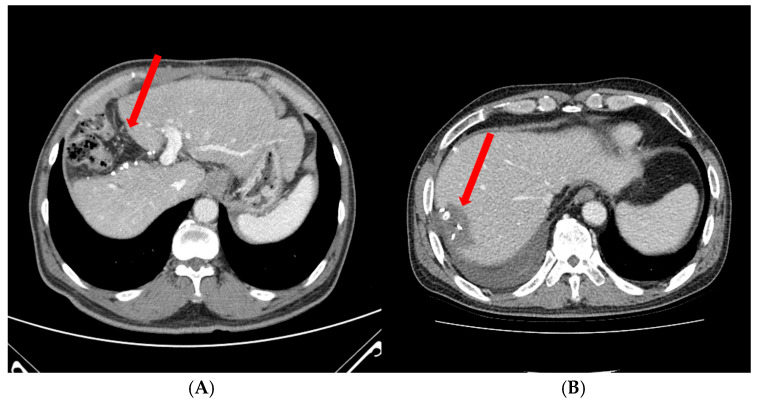
Anatomical (**A**) and non-anatomical (**B**) resection for HCC. Red arrows indicate the resection surface after hepatectomy.

Liver resection is a primary treatment for HCC, aiming to remove the tumor while preserving functional liver tissue [7]. The debate between anatomical resection (AR) and non-anatomical resection (NAR) has been ongoing in the field of HCC surgery. AR involves removal of the tumor along with the portal territories that drain it, based on the concept that HCC tends to spread along portal tributaries. This approach has shown improved recurrence-free survival compared to NAR [32]. Several studies have demonstrated improved long-term outcomes with AR, particularly for solitary HCC less than 5 cm in size [33]. Figure 1A shows an AR performed for curative resection of HCC.

NAR, on the other hand, involves the removal of the tumor with a margin of surrounding liver tissue, without strict adherence to segmental anatomy. This approach can be particularly useful for peripheral small tumors or in situations where the preservation of liver parenchyma is crucial. Some studies have shown comparable outcomes between AR and NAR, especially for small HCC [34]. Figure 1B shows NAR performed for a peripheral small-sized HCC.

A recent meta-analysis summarized 22 propensity score-matched studies that compared AR and NAR [35]. This meta-analysis, comprising 2496 cases of AR and 2590 cases of NAR, demonstrated superior oncological outcomes with AR for HCC. AR was associated with better 3-year and 5-year overall survival, as well as improved recurrence-free survival at 1, 3, and 5 years, with notably lower rates of local and multiple intrahepatic recurrence. These benefits were particularly pronounced in tumors ≤5 cm and in patients with microscopic spread. While AR showed comparable postoperative complications to NAR, its survival advantage was less evident in cirrhotic patients, where both approaches demonstrated similar recurrence-free survival rates at 3 and 5 years.

The choice between these approaches often depends on tumor location and size, underlying liver function, and surgeon preference. Some studies suggest that the extent of resection margin may be more important than the anatomical versus non-anatomical distinction, with a margin width of at least 1 cm associated with better outcomes [36].

Postoperative CT findings follow two different surgical approaches for hepatocellular carcinoma (HCC). Panel A illustrates an anatomical resection, where entire liver segment(s) containing the tumor are removed along anatomical planes. The CT image shows a defined, straight resection margin corresponding to anatomical boundaries, with complete removal of the affected portal venous and hepatic arterial branches. Panel B depicts the results of a non-anatomical resection, where the tumor is removed with a margin of surrounding liver tissue, irrespective of segmental anatomy. The CT image shows an irregular resection margin and potential preservation of nearby liver segments.

### 4.2. Parenchyma-Sparing Techniques

Parenchyma-sparing techniques have become increasingly important in HCC resection, particularly given the high rate of recurrence and the potential need for future resections. These techniques aim to remove the tumor with an adequate margin while preserving as much functional liver tissue as possible. Approaches include wedge resections, enucleations (for well-circumscribed tumors), and limited segmentectomies.

Advanced imaging techniques play a crucial role in these approaches. Intraoperative contrast-enhanced ultrasonography aids in precise diagnosis and surgical guidance [32]. Other techniques, such as indocyanine green fluorescence imaging, can aid in identifying segmental boundaries and ensuring adequate margins [37].

Parenchyma-sparing resection is particularly valuable in patients with limited liver reserve or those with multifocal disease who may require multiple resections over time. For tumors ≤ 5 cm, resection and combined TACE with radiofrequency ablation rank highest for survival outcomes [38].

### 4.3. Extended Hepatectomy Considerations

Extended hepatectomy, defined as resection of five or more liver segments, may be necessary for large or multifocal HCC. However, this approach carries a significant risk of post-hepatectomy liver failure, particularly in patients with underlying liver disease. Careful patient selection is crucial, with the assessment of future liver remnant (FLR) volume and function being paramount.

When the anticipated FLR is inadequate, techniques such as portal vein embolization (PVE) or associating liver partition and portal vein ligation for staged hepatectomy (ALPPS) may be employed to induce liver hypertrophy preoperatively [39,40]. These techniques can increase the pool of patients eligible for extended resection, but they also carry their own risks and should be employed judiciously.

ALPPS represents an innovative two-stage surgical approach that addresses the limitations of traditional PVE and portal vein ligation (PVL). Unlike conventional methods that require 8–12 weeks and achieve only 20–40% hypertrophy, ALPPS induces rapid liver hypertrophy of up to 90% within 6–10 days [41]. This technique offers several advantages: it allows for aggressive future liver remnant clean-up, achieves superior hypertrophy rates, reduces adhesion formation due to shorter intervals between stages, enables single hospitalization, and demonstrates higher resectability rates compared to traditional approaches.

While initial concerns about high morbidity and mortality rates prompted careful consideration, subsequent evidence suggests that ALPPS can be performed safely in experienced centers, particularly for patients with metastatic liver disease under 60 years of age. The technique has proven especially valuable in two specific scenarios: as a rescue option for failed portal vein occlusion and in cases of extensive bilateral disease where extremely small future liver remnants might result from tumor clean-up. Although ALPPS is unlikely to replace PVE for patients with tumor-free future liver remnants, it may supersede classic two-stage resections in selected cases, pending evidence of comparable long-term outcomes.

PVE has emerged as a crucial preoperative strategy for patients undergoing major hepatic resection, particularly when the estimated FLR is insufficient (less than 25–40% of total liver volume) or in patients with underlying liver disease. The procedure, which preferably utilizes cyanoacrylate glue as the embolic agent, induces compensatory hypertrophy of the non-embolized segments, with maximal growth occurring in the first two weeks and continuing for approximately six weeks. This selective hypertrophy approach has multiple benefits: it reduces postoperative complications, shortens hospital stays, and expands the pool of surgical candidates who were initially deemed unsuitable for resection [42]. The success of the procedure requires a thorough understanding of hepatic segmental anatomy, proper patient selection, careful timing of subsequent resection (typically performed 2–6 weeks post-embolization), and awareness of potential complications.

Recent advances have expanded indications for resection to include larger tumors and those with vascular invasion [43]. However, the decision to proceed with extended hepatectomy must be carefully balanced against the risks of postoperative liver failure and the potential for alternative treatments.

### 4.4. Laparoscopic vs. Open Approaches

In recent years, laparoscopic liver resection (LLR) has gained popularity as an alternative to the traditional open liver resection (OLR) for HCC. LLR offers several potential advantages, including reduced blood loss, shorter hospital stay, and faster recovery [44]. Initially limited to wedge resections and left lateral sectionectomies, laparoscopic techniques have now been extended to major hepatectomies in experienced centers.

Meta-analyses have been reported, summarizing the results of many studies that analyzed LLR versus OLR for HCC. A study reported in 2017 by Sotiropoulos et al., encompassing 5203 patients from 44 studies, demonstrated that LLR was associated with reduced blood loss, lower transfusion requirements, better R0 resection rates, wider resection margins, shorter hospital stays, and reduced morbidity and 30-day mortality rates compared to OLR [45]. Similarly, Wang et al.’s focused analysis of elderly patients (≥65 years), reported in 2022 with 1346 patients, showed that despite longer operative times, LLR resulted in significantly shorter hospital stays and fewer overall and severe postoperative complications [46]. Notably, both meta-analyses found no significant differences in oncological outcomes, including tumor recurrence, overall survival, and disease-free survival rates between the two approaches, suggesting that LLR is not only safer but also oncologically equivalent to OLR, particularly for small HCC and in elderly patients.

However, LLR is technically challenging and has a relatively long learning curve. It may be particularly difficult in patients with cirrhosis or for tumors in posterior or superior liver segments. The choice between laparoscopic and open approaches depends on tumor location, extent of resection required, surgeon expertise, and patient factors. Despite the advantages of laparoscopic surgery, open resection remains the standard approach for complex cases or when extensive resection is required.

### 4.5. Hanging Maneuver

The liver hanging maneuver is a widely used surgical technique that enhances the safety and efficiency of hepatic resection procedures. This approach involves suspending the liver using tape positioned between the inferior vena cava and the liver tissue, which provides better exposure and control during parenchymal division [47]. Since its introduction, the technique has evolved to accommodate various types of liver resections and has demonstrated increasing success rates, now reaching 94% [48]. While anatomical variations and adhesions can sometimes limit its application, the hanging maneuver has consistently shown benefits in minimizing blood loss and reducing surgical complications during liver resection procedures [49].

### 4.6. Robotic-Assisted Surgery

Robotic-assisted surgery represents a significant technological leap in the field of HCC resection. While laparoscopic approaches have gained popularity due to their minimally invasive nature, they come with technical challenges, such as limited range of motion and reduced tactile feedback. Robotic systems aim to address these limitations while maintaining the benefits of minimally invasive surgery.

Robotic platforms, such as the da Vinci Surgical System, offer several advantages for HCC resection. These include enhanced three-dimensional visualization, increased dexterity with wristed instruments that can rotate 360 degrees, and improved ergonomics for the surgeon. These features can be particularly beneficial in complex hepatectomies or in accessing difficult-to-reach areas of the liver.

Early studies on robotic-assisted HCC resection have shown promising results. A systematic review by Montalti et al. (2016) found that robotic liver resections were associated with low conversion rates to open surgery, minimal blood loss, and short hospital stays [50]. However, the authors noted that most reported cases were minor hepatectomies, and more data are needed on major hepatectomies and long-term oncological outcomes.

The integration of robotic systems with other advanced technologies, such as intraoperative imaging and navigation systems, opens up new possibilities for precision in HCC resection. For instance, some systems allow for real-time overlay of preoperative imaging data onto the surgical field, enhancing the surgeon’s ability to navigate complex anatomy [51].

The advantages and disadvantages of open, laparoscopic and robotic approaches for surgical resection of HCC are summarized in Table 2.

### 4.7. Conclusions

The choice of resection strategy for HCC is complex and must be tailored to each individual patient. Factors to consider include tumor characteristics, liver function, underlying liver disease, and surgeon expertise. While anatomical resection has traditionally been preferred, there is increasing recognition of the value of parenchyma-sparing approaches, particularly in the context of cirrhosis and potential future recurrence. Extended hepatectomies may be necessary for advanced tumors but carry significant risks. Laparoscopic and robotic techniques offer potential benefits in terms of reduced morbidity but require significant expertise. As our understanding of HCC biology and surgical techniques continues to evolve, ongoing research focuses on refining patient selection criteria, improving surgical techniques, and integrating resection with other treatment modalities to optimize outcomes for patients with HCC.

## 5. Emerging Technologies in HCC Resection

The landscape of HCC management is rapidly evolving, with emerging technologies revolutionizing traditional approaches to surgical resection. These advancements aim to enhance surgical precision, minimize invasiveness, and improve patient outcomes. Recent years have seen the expansion of treatment options beyond conventional surgical resection and transplantation, encompassing a range of innovative techniques and technologies. This section explores two key areas of technological innovation in HCC resection: 3D printing and surgical planning, and intraoperative navigation systems. As these technologies continue to develop and integrate into clinical practice, they promise to reshape the landscape of HCC surgical management, offering new hope for improved patient care and outcomes.

### 5.1. 3D Printing and Surgical Planning

Three-dimensional (3D) printing technology has emerged as a valuable tool in preoperative planning for HCC resection. This technology allows for the creation of patient-specific, tangible 3D models of the liver, tumors, and surrounding vasculature based on CT or MRI imaging data. These models provide surgeons with a tactile and visual representation of the patient’s unique anatomy, allowing for detailed preoperative planning and simulation of the surgical approach.

Studies have shown that 3D-printed models can enhance surgeons’ understanding of spatial relationships between tumors and critical structures, potentially leading to more precise resections and reduced operative times [52]. For instance, a study by Witowski et al. (2019) reported that the use of 3D-printed liver models in preoperative planning for complex HCC cases resulted in changes to the surgical plan in 33% of cases, demonstrating the technology’s potential to influence decision making and improve surgical outcomes [53].

### 5.2. Intraoperative Navigation Systems

Intraoperative navigation systems represent another significant advancement in HCC resection technology. These systems integrate preoperative imaging data with real-time intraoperative imaging, allowing surgeons to navigate through the liver with enhanced precision and confidence.

Typically, these systems use a combination of preoperative CT or MRI images, intraoperative ultrasound, and optical tracking technology. The preoperative images are registered to the patient’s anatomy in the operating room, and the surgeon can then track the position of surgical instruments in relation to the patient’s anatomy in real time [54].

One of the key advantages of these systems is their ability to compensate for liver deformation and movement during surgery. Advanced navigation systems use deformable registration algorithms to update the virtual model of the liver in real time, maintaining accuracy throughout the procedure [55].

Intraoperative navigation has shown particular promise in laparoscopic liver resections, where tactile feedback is limited. These systems can help surgeons to register and visualize the preoperatively planned resection plane [56].

The development of intraoperative fluorescence guidance techniques has further enhanced the precision of HCC resection. Indocyanine green fluorescence imaging can be used to evaluate tumor differentiation and fibrosis in the non-cancerous liver parenchyma during hepatocellular carcinoma resection [57].

### 5.3. Conclusions

The emergence of advanced technologies in HCC resection, including 3D printing for surgical planning and intraoperative navigation systems, represents a significant step forward in the field. These technologies offer the potential for more precise preoperative planning and enhanced intraoperative guidance, ultimately improving surgical execution.

While these technologies show great promise, it is important to note that they are still evolving, and their impact on long-term patient outcomes is still being evaluated. Moreover, challenges remain in treating advanced HCC, and there is a need for well-controlled comparative trials to guide treatment decisions [58]. As research continues and these technologies mature, they are likely to play an increasingly important role in improving the precision and safety of HCC resections, ultimately leading to better patient outcomes.

## 6. Perioperative Management

The perioperative management of patients undergoing HCC resection is a critical component of the overall treatment strategy. It encompasses a wide range of considerations, from preoperative assessment and anesthetic management to postoperative care and complication prevention. Effective perioperative management can significantly impact patient outcomes, potentially reducing morbidity and mortality rates and improving long-term survival. This section will focus on two key aspects of perioperative management: anesthesia considerations and postoperative care and complications.

### 6.1. Anesthesia Considerations

Preoperative assessment of liver function and comorbidities is essential for patient selection and risk stratification [59,60]. This evaluation typically includes liver function tests, coagulation profiles, and the assessment of portal hypertension. The Child-Pugh score and MELD score are commonly used to evaluate liver function and predict perioperative risk [61].

Anesthetic considerations for HCC patients are crucial due to the complex interplay between anesthesia, tumor biology, and patient outcomes. Different anesthetic techniques may influence cancer progression and recurrence [62]. The choice of anesthetic approach must take into account not only the immediate surgical requirements but also potential long-term oncological implications. While various anesthetic methods can be used for HCC procedures, total intravenous anesthesia with propofol and thoracic epidural analgesia may be preferred for liver resections [63]. This approach may offer benefits in terms of postoperative pain control. However, the choice of anesthetic technique remains a subject of ongoing research and debate. Interestingly, a retrospective study found that general anesthesia was associated with reduced cancer recurrence compared to epidural anesthesia in percutaneous radiofrequency ablation [64]. This finding underscores the potential impact of anesthetic choices on long-term oncological outcomes.

### 6.2. Postoperative Care and Complications

Postoperative management of HCC requires careful consideration of various factors. The immediate postoperative period is critical, with a focus on maintaining hemodynamic stability, ensuring adequate liver function, and preventing complications.

Accurate preoperative evaluation of liver function, nutrition, and inflammation is crucial for predicting and managing postoperative outcomes. Patients with poor preoperative nutritional status or significant inflammation may be at higher risk for complications and may benefit from preoperative optimization strategies.

The early implementation of enhanced recovery programs can reduce morbidity and accelerate recovery [65]. These programs typically include elements, such as early mobilization, early enteral nutrition, and multimodal pain management. Enhanced recovery after surgery (ERAS) protocols have been found to significantly reduce the length of hospital stay and overall complications in patients undergoing liver resection for HCC [66].

Proper patient selection based on tumor characteristics and physical reserve is essential for optimal surgical outcomes [60]. While hepatic resection and liver transplantation remain primary curative options, local ablative therapies may offer comparable survival for small HCCs [67]. The choice of treatment modality should be individualized based on tumor size, location, and patient factors.

Postoperative recurrence is a significant concern, with pathologic factors like venous invasion and tumor size being established risk factors [68]. Close postoperative surveillance is essential for the early detection of recurrence. According to the current guidelines proposed by the European Association for the Study of the Liver, follow-up strategies for detecting recurrence recommend contrast-enhanced computed tomography (CT) scans every 3 months during the first year, followed by every 6 months afterward [69]. However, Lee et al. proposed a more individualized approach in their retrospective analysis of 1316 patients [70]. They concluded that for high-risk patients, conducting surveillance every 3 months can extend survival compared to a 6-month interval. However, for low-risk patients, 3-monthly surveillance may not offer any survival advantage over 6-monthly surveillance.

The optimal management strategy for recurrence depends on factors such as the pattern of recurrence, liver function, and patient performance status.

Adequate nutritional support, glycemic control, and measures to reduce infectious complications are crucial for improving postoperative outcomes [71]. Malnutrition is common in patients with HCC and cirrhosis and has been associated with increased postoperative complications and mortality. Implementing strategies to optimize nutritional status, both pre- and postoperatively, can significantly impact outcomes [72].

Post-hepatectomy liver failure (PHLF) remains one of the most serious complications following HCC resection. In 2005, Balzan et al. were the first to systematically describe clinically relevant liver failure. By analyzing blood samples (bilirubin and prothrombin time) on postoperative day five, they were able to predict 60-day mortality [73]. In 2010, Mullen et al. introduced another definition, focusing solely on peak bilirubin levels during the postoperative period [74]. In 2011, the International Study Group of Liver Surgery (ISGLS) established a definition based on an expert consensus, outlining three grades of PHLF (A–C) [75]. This grading system is based on bilirubin and prothrombin time measurements taken on or after postoperative day five, along with the patient’s clinical progression after hepatectomy. There have been recent attempts to refine the prediction of PHLF using methods such as perioperative lactate dynamics, but these attempts have not resulted in widely accepted definitions [76]. Currently, the ISGLS PHLF criteria are the most frequently used in the literature to define PHLF. Early identification and aggressive management of liver failure are critical for improving outcomes.

### 6.3. Conclusions

Effective perioperative management is crucial for optimizing outcomes in patients undergoing HCC resection. Anesthetic considerations must balance immediate surgical needs with potential long-term oncological impacts. The choice of anesthetic technique, based on patient factors and emerging research on anesthesia-related genes, may influence cancer recurrence and progression. Postoperative care focuses on preventing and managing complications, with particular attention to liver function, nutrition, and early mobilization. Enhanced recovery programs have shown promise in improving postoperative outcomes. Long-term management strategies must address the risk of tumor recurrence through close surveillance and appropriate interventions. As our understanding of the complex interplay between perioperative factors and HCC outcomes continues to evolve, personalized approaches to perioperative management may further improve patient outcomes.

## 7. Outcomes and Prognostic Factors

Hepatic resection for HCC remains a cornerstone of curative treatment, offering the potential for long-term survival in selected patients. However, the outcomes of surgical resection are influenced by a complex interplay of factors, including tumor characteristics, liver function, surgical techniques, and patient-specific variables. Understanding these outcomes and the factors that influence them is crucial for patient selection, treatment planning, and prognostication. This section will explore the short-term outcomes, long-term outcomes, and factors influencing prognosis in HCC resection.

### 7.1. Short-Term Outcomes (Morbidity, Mortality)

Surgical resection for HCC remains a primary treatment option, but it carries significant risks. Morbidity rates range from 10.5% to 35.1%, while mortality rates vary from 1.0% to 8.3% [77,78]. These wide ranges reflect the heterogeneity of patient populations, tumor characteristics, and institutional experiences.

Risk factors for complications include prolonged operating time, bile leakage, cirrhosis, excessive blood loss, and malnutrition [72,79,80]. The extent of liver resection is particularly crucial, with major hepatectomies associated with higher complication rates compared to minor resections. A study by Cucchetti et al. (2012) found that the risk of post-hepatectomy liver failure increases significantly when the future liver remnant volume is less than 30% in patients with normal liver function, or less than 40% in those with compromised liver function [81].

Despite these risks, resection offers better long-term survival compared to alternatives like transarterial chemoembolization [82]. This underscores the importance of careful patient selection to balance the potential benefits against the risks of surgical intervention.

For early-stage HCC, parenchymal-sparing resections or ablation may be preferable to major hepatectomy due to lower complication rates [77,83]. The advent of laparoscopic and robotic approaches has also contributed to reduced morbidity in selected cases. A systematic review by Ciria et al. (2015) found that LLR for HCC was associated with lower complication rates and shorter hospital stays compared to open surgery, without compromising oncological outcomes [44].

### 7.2. Long-Term Outcomes (Recurrence, Survival)

Recurrence after surgical resection remains a significant challenge, occurring in 47–70% of patients [84,85,86]. This high recurrence rate underscores the importance of long-term surveillance and management strategies.

Factors associated with recurrence and survival include tumor characteristics, liver function, and treatment modalities [87,88]. Tumor size, number, vascular invasion, and differentiation grade are particularly important predictors of recurrence. A meta-analysis by Rodríguez-Perálvarez et al. (2013) found that microvascular invasion was associated with a nearly three-fold increase in the risk of recurrence [89].

Despite high recurrence rates, aggressive management of recurrence can improve long-term outcomes. Strategies for managing recurrence include repeat resection, ablation, and systemic therapies. A study by Xu et al. (2019) demonstrated that repeat hepatectomy for recurrent HCC could achieve 5-year overall survival rates of up to 56%, comparable to those of primary resection [90].

Global 5-year overall survival rates following resection are approximately 56%, with higher rates observed in Asia compared to other regions [91]. This geographic variation may be attributed to differences in etiological factors, surveillance practices, and treatment strategies. Recurrence-free survival at 5 years is around 35%, highlighting the persistent challenge of tumor recurrence [91].

Prognostic models and nomograms have been developed to predict recurrence and survival, aiding in patient counseling and treatment planning [87,88]. These tools incorporate various clinicopathological factors to provide individualized risk estimates. For instance, the Barcelona Clinic Liver Cancer (BCLC) staging system has been widely adopted for its ability to link staging with treatment recommendations and prognostic predictions [92].

Ongoing research focuses on improving long-term outcomes through enhanced surveillance, recurrence prevention, and novel therapeutic approaches [93]. The integration of molecular markers and genomic profiling holds promise for more precise prognostication and personalized treatment strategies. A study by Hoshida et al. (2013) identified a gene expression signature in the liver microenvironment that was predictive of late recurrence, suggesting potential targets for chemoprevention [94].

### 7.3. Factors Influencing Prognosis

Surgical resection for HCC has varying prognostic outcomes influenced by multiple factors. These can be broadly categorized into tumor-related, liver-related, and treatment-related factors.

Tumor characteristics, such as size >5 cm, multifocality, vascular invasion, and high alpha-fetoprotein (AFP) levels, are associated with poor prognosis [95,96,97]. A meta-analysis by Zhang et al. (2014) confirmed that microvascular invasion was a significant predictor of both overall survival and disease-free survival following HCC resection [98].

Surgical factors, including blood loss and transfusion, negatively impact disease-free and overall survival, particularly in larger tumors [99]. This underscores the importance of meticulous surgical techniques and blood conservation strategies. The extent of resection margin has also been shown to influence outcomes, with wider margins associated with improved survival in some studies [100].

Preoperative imaging findings can provide valuable prognostic information. High standardized uptake value on PET-CT and apparent diffusion coefficient on MRI can predict outcomes [101,102]. These imaging biomarkers may reflect tumor biology and aggressiveness, offering additional tools for risk stratification.

Liver-related factors play a crucial role in determining prognosis. Child-Pugh class, cirrhosis severity, and liver function markers significantly affect outcomes [103]. The presence of portal hypertension, in particular, has been associated with poorer outcomes following resection [104].

Emerging factors influencing prognosis include molecular and genetic markers. For instance, mutations in the TERT promoter have been associated with increased risk of recurrence following resection [105]. The integration of these molecular markers with traditional clinicopathological factors holds promise for more precise prognostication and personalized treatment strategies.

### 7.4. Conclusions

The outcomes of HCC resection are influenced by a complex interplay of tumor characteristics, liver function, surgical factors, and patient-specific variables. Short-term outcomes have improved over time due to advances in surgical techniques, perioperative management, and patient selection. However, long-term outcomes remain challenged by high recurrence rates. Prognostic factors span a wide range, from tumor biology to liver function and surgical approach. The integration of these factors into staging systems and prognostic models aids in treatment planning and patient counseling. As our understanding of HCC biology deepens and treatment modalities evolve, there is hope for continued improvement in both short-term and long-term outcomes. Future research directions include refining prognostic models, developing strategies to prevent recurrence, and personalizing treatment approaches based on individual patient and tumor characteristics.

## 8. Conclusions

### 8.1. Summary of Key Points

HCC remains a significant global health challenge, with surgical resection continuing to play a crucial role in its management. This review explored various aspects of HCC resection, from preoperative considerations to long-term outcomes. Key points that have emerged include the importance of careful patient selection, the evolving nature of resection strategies, and the impact of emerging technologies on surgical outcomes.

Current guidelines for HCC resection emphasize the need for a multidisciplinary approach, with patient selection criteria considering tumor characteristics, liver function, and overall health status. The debate between anatomical and non-anatomical resection continues, with recent evidence suggesting that the extent of resection margin may be more critical than the specific approach. Parenchyma-sparing techniques have gained prominence, particularly for patients with limited liver reserve or those at risk of recurrence.

Laparoscopic and robotic-assisted approaches have significantly impacted the field of HCC resection. These minimally invasive techniques have shown promise in reducing perioperative morbidity while maintaining oncological outcomes. Laparoscopic resection, now extended to major hepatectomies in experienced centers, offers benefits such as reduced blood loss and shorter hospital stays. Robotic-assisted surgery provides enhanced three-dimensional visualization and increased dexterity, potentially improving precision in complex cases. However, these advanced techniques require significant expertise and may not be universally applicable, highlighting the importance of proper training and patient selection.

Emerging technologies have further revolutionized HCC resection practices. Three-dimensional printing and advanced imaging techniques have enhanced preoperative planning and intraoperative guidance. Intraoperative navigation systems, integrating preoperative imaging with real-time data, allow for more precise tumor localization and resection. These technologies offer the potential for improved surgical accuracy and outcomes, particularly in challenging cases.

Perioperative management remains critical in optimizing outcomes. Anesthetic considerations must balance immediate surgical needs with potential long-term oncological impacts. Enhanced recovery after surgery (ERAS) protocols have demonstrated benefits in reducing complications and hospital stay. Postoperative care focuses on preventing and managing complications, with particular attention to liver function and early mobilization.

Long-term outcomes following HCC resection have improved over time, but recurrence remains a significant challenge. Factors influencing prognosis span a wide range, from tumor biology to liver function and surgical approach. The integration of these factors into staging systems and prognostic models aids in treatment planning and patient counseling. Aggressive management of recurrence, including repeat resection and ablation, has shown promise in improving long-term survival.

### 8.2. Remaining Challenges and Research Needs

Despite the progress made in HCC resection, several challenges remain, presenting opportunities for future research. One of the most pressing issues is the high rate of tumor recurrence following resection. While we have identified several risk factors for recurrence, our understanding of the underlying biological mechanisms remains incomplete. Research is needed to develop more effective strategies for preventing recurrence, potentially through adjuvant therapies or targeted interventions based on individual tumor biology.

The optimal approach to resection in patients with cirrhosis or portal hypertension remains controversial. Further studies are required to refine selection criteria and develop strategies to expand the pool of eligible candidates without compromising outcomes. This may involve novel preoperative optimization techniques or intraoperative strategies to preserve liver function.

The role of minimally invasive approaches, including laparoscopic and robotic-assisted resection, needs further clarification. While these techniques have shown promise, long-term oncological outcomes and cost-effectiveness require more extensive evaluation. Research should focus on identifying which patients are most likely to benefit from these approaches and developing standardized training protocols to ensure consistent outcomes across centers.

The integration of molecular and genetic markers into prognostic models and treatment decision making represents an exciting frontier. While some molecular signatures have shown promise in predicting recurrence and survival, their clinical utility remains limited. Large-scale, prospective studies are needed to validate these markers and develop practical tools for their implementation in clinical practice.

The impact of systemic therapies, including immunotherapy and targeted agents, in the perioperative setting is an area of active investigation. Research is needed to determine the optimal timing and sequencing of these therapies in relation to surgical resection, as well as their potential role in preventing recurrence.

Finally, there is a need for more robust comparative studies between resection and other treatment modalities, particularly for early-stage HCC. This includes comparisons with liver transplantation, ablation techniques, and emerging locoregional therapies. Such studies should consider not only survival outcomes but also quality of life and cost-effectiveness.

In conclusion, while surgical resection remains a cornerstone in the management of HCC, there is significant room for improvement. Future research should focus on personalizing treatment approaches based on individual patient and tumor characteristics, developing more effective strategies to prevent and manage recurrence, and integrating emerging technologies and therapies to optimize both short-term and long-term outcomes. As our understanding of HCC biology deepens and treatment modalities evolve, the field of HCC resection is poised for continued advancement, offering hope for improved outcomes for patients facing this challenging disease.

## Figures and Tables

**Table 1:** Comparison of major international guidelines for hepatocellular carcinoma resection.

Guideline	AASLD	EASL	APASL	JSH
Indications for Resection	Early-stage HCC with preserved liver function, single tumors, and absence of portal hypertension.	Resectable tumors with preserved liver function (Child-Pugh A), no portal hypertension, and sufficient future liver remnant.	Child-Pugh A or selected B patients with single or few nodules; absence of significant portal hypertension.	Suitable for patients with good liver function (Child-Pugh A) and no severe portal hypertension.
Assessment of Liver Function	Child-Pugh score, MELD score, and portal hypertension evaluation.	Child-Pugh score and presence of clinically significant portal hypertension.	Child-Pugh classification, MELD score, and ICG-R15 test.	Child-Pugh score, ICG clearance test, and liver stiffness measurement.
Tumor Size and Number	Typically single tumors; some centers may consider resection for limited multifocal disease.	Single or multiple tumors without macrovascular invasion and good liver function.	Single tumors are preferred; selected cases of limited multifocal tumors.	Single tumors are preferred; some selected multifocal cases are considered.
Portal Hypertension Considerations	Contraindicated if significant portal hypertension is present.	Contraindicated in patients with clinically significant portal hypertension.	Generally contraindicated in the presence of significant portal hypertension; exceptions in highly selected cases.	Resection not recommended for patients with severe portal hypertension; mild cases may be considered.
Future Liver Remnant (FLR)	Adequate FLR is crucial; FLR volume usually ≥30–40% for safe resection.	Sufficient FLR required; strategies like portal vein embolization may be considered.	Emphasizes adequate FLR; preoperative portal vein embolization considered for small FLR.	FLR evaluation is crucial; often recommends ≥ 30% FLR volume post-resection.
Role of Preoperative Therapies	Limited role; neoadjuvant therapies not typically recommended prior to resection.	Preoperative locoregional therapies considered in downstaging or bridging to resection in select cases.	Downstaging considered if tumor burden is reduced to resectable limits.	Preoperative TACE or ablation considered in select cases for tumor reduction.
Surgical Margins	Negative margins recommended, though no clear consensus on width.	Negative margins preferred; margin width not strictly defined.	Advocates for R0 resection; margin width not strictly defined.	Negative margins (R0 resection) are the goal; wider margins preferred when feasible.
Minimally Invasive Surgery	Laparoscopic or robotic surgery considered for select patients with favorable anatomy and tumors.	Laparoscopic resection encouraged for select patients with small, peripheral tumors and preserved liver function.	Minimally invasive surgery favored in select cases with small, accessible tumors.	Laparoscopic and robotic approaches are preferred for early-stage, small HCC with peripheral location.
Postoperative Surveillance	Regular surveillance with imaging (CT/MRI) and AFP monitoring every 3–6 months.	Close surveillance with imaging and AFP levels every 3–6 months post-resection.	Imaging and AFP monitoring every 3–6 months, adjusting frequency based on recurrence risk.	Regular surveillance with imaging and tumor markers every 3–4 months.

**Table 2:** Advantages and disadvantages for open, laparoscopic and robotic approaches for surgical resection of hepatocellular carcinoma.

Criteria	Open Surgery	Laparoscopic Surgery	Robotic Surgery
Pros	Standard for complex and extensive resections.	Reduced blood loss.	Enhanced 3D visualization and increased dexterity.
	No limitations on tumor location or size.	Shorter hospital stay and faster recovery.	Improved ergonomics for the surgeon.
	No technical constraints regarding access or feedback.	Comparable oncological outcomes to open surgery.	Minimal blood loss and low conversion rates.
			Integration with advanced imaging and navigation.
Cons	Longer hospital stay and recovery time.	Technically challenging with a steep learning curve.	High costs associated with robotic systems.
	Greater blood loss.	Difficult in cirrhotic patients or certain tumor locations.	Limited data on major resections and outcomes.
	Higher risk of postoperative complications.	Limited by surgeon experience and tumor location.	Predominantly used for minor resections.
			Limited availability in many centers.
